# Retrospective Serology Study of Respiratory Virus Infections in Captive Great Apes

**DOI:** 10.3390/v6031442

**Published:** 2014-03-24

**Authors:** Hester Buitendijk, Zahra Fagrouch, Henk Niphuis, Willy M. Bogers, Kristin S. Warren, Ernst J. Verschoor

**Affiliations:** 1 Department of Virology, Biomedical Primate Research Centre (BPRC), Lange Kleiweg 161, 2288GJ, Rijswijk, The Netherlands; E-Mails: buitendijk@bprc.nl (H.B.); fagrouch@bprc.nl (Z.F.); niphuis@bprc.nl (H.N.); bogers@bprc.nl (W.M.B.); 2 School of Veterinary and Life Sciences, Murdoch University, Murdoch, 6150, Western Australia, Australia; E-Mail: k.warren@murdoch.edu.au

**Keywords:** respiratory viruses, great apes, serology, anthroponotic infections

## Abstract

Great apes are extremely sensitive to infections with human respiratory viruses. In this study, we retrospectively analyzed sera from captive chimpanzees, gorillas and orang-utans. More than 1000 sera (403 chimpanzee, 77 gorilla, and 535 orang-utan sera) were analyzed for antibodies to the human respiratory viruses RSV (respiratory syncytial virus, hMPV (human metapneumovirus), H1N1 and H3N2 influenza A viruses, and influenza B virus. In all ape species high seroprevalences were found for RSV, hMPV, and influenza B virus. A high percentage of captive chimpanzees also showed evidence of influenza A H1N1 infections, and had low levels of H3N2 antibodies, while in sera from gorillas and orang-utans antibody levels to influenza A and B viruses were much lower or practically absent. Transmission of respiratory viruses was examined in longitudinal sera of young chimpanzees, and in chimpanzee sera taken during health checks. In young animals isolated cases of influenza infections were monitored, but evidence was found for single introductions followed by a rapid dissemination of RSV and hMPV within the group. Implementation of strict guidelines for handling and housing of nonhuman primates was shown to be an efficient method to reduce the introduction of respiratory infections in colonies of captive animals. RSV seroprevalence rates of chimpanzees remained high, probably due to circulating virus in the chimpanzee colony.

## 1. Introduction

The transfer of nonhuman primate viruses to humans in the past and present has been extensively documented [1,2]. Most notable is the transmission of simian immunodeficiency virus from chimpanzees, gorillas, and sooty mangabeys that started the AIDS pandemic, but other widely known human viruses, like dengue virus, Chikungunya virus and human T-cell lymphotropic virus (HTLV), also have their origin in nonhuman primates [3,4,5,6,7]. Additionally, infections of humans with nonhuman primate viruses, like herpes B virus, Ebola virus, monkey poxvirus, and foamy viruses have also been described [8,9,10,11]. 

Conversely, nonhuman primates also run the risk of anthroponotic, i.e., from human to nonhuman primate, infections. Measles virus and herpesviruses, such as herpes simplex virus 1 (HSV-1) and varicella zoster virus, are examples of viruses that are able to jump the species barrier, and cause outbreaks in colonies of captive animals [12,13,14]. Particularly, great apes are extremely vulnerable to several human viral diseases. In recent years, several reports have been published detailing human respiratory viruses infecting wild African apes. Viruses like human metapneumovirus (hMPV), respiratory syncytial virus (RSV), or influenza viruses have repeatedly caused outbreaks of flu-like disease with high morbidity, and deaths amongst chimpanzees and gorillas have been attributed to infections with these pathogens [15].

Obviously, groups of captive great apes are equally vulnerable to these human viruses, and due to the regular and close contacts with animal caretakers or veterinary staff, one would expect infections to occur more commonly. However, publications on this topic are quite rare. Kilbourn *et al.* [16] performed a survey amongst 84 free-ranging and 60 semi-captive orangutans for evidence of infection with 47 different viruses, including RSV and influenza A and B viruses. They found serological evidence for RSV infections in two animals (1.4%), but did not detect antibodies to the other respiratory viruses. Recently, Kooriyama *et al.* [17] investigated sera from 14 captive chimpanzees for evidence of infection with 63 pathogens, including respiratory viruses. RSV and hMPV antibodies were detected in all animals, influenza A H3N2 was identified in one animal, while H1N1 and influenza B virus infections were absent. Finally, Unwin *et al.* [18] reported an acute outbreak of RSV in a group of 30 captive chimpanzees.

To extend our knowledge on the prevalence and transfer of human respiratory viruses to captive apes, we investigated three species of great apes for antibodies to four common respiratory viruses: hMPV, RSV, influenza A virus, and influenza B virus. The animals had different backgrounds: the chimpanzee sera were obtained from the former colony of Western common chimpanzees that was housed at the Biomedical Primate Research Centre (BPRC) in Rijswijk, the Netherlands; the gorilla sera had been sampled from animals living in various zoos; and all orangutan sera were collected from apes that were housed at the Wanariset Orangutan Rehabilitation Centre in East Kalimantan, Indonesia.

## 2. Results

### 2.1. Sera

The sera tested in this study were obtained from different sources. We analyzed 403 serum samples from 203 individual chimpanzees that were housed at the Biomedical Primate Research Centre (BPRC) in Rijswijk, the Netherlands. Additional sera were obtained at the regular health examinations from a group of young animals. The gorilla sera (n = 77) were all derived from zoo animals. The orangutan sera (535 sera from 179 individuals) had been sampled from animals that were housed at the Wanariset Rehabilitation Orang-utan Centre in East Kalimantan, Indonesia, in the period from 1994 to 1998. 

### 2.2. Serological Survey of Respiratory Infections in Great Ape Species

Sera were analyzed by using an in-house developed magnetic bead-based multiplex assay for the presence of antibodies to RSV, hMPV, influenza A virus, and influenza B virus. Antibodies which were reactive to the influenza A virus strain H3N2 Texas 1/77 and the pandemic H1N1 influenza strain California/7/2009 were measured separately. Results were confirmed with Western blot using the same purified viral antigens and infected cell-lysates. A stringent cut-off rate equal to four times the average background signal was used to avoid false-positive results due to the variable quality of the sera. The seroprevalence rates of specific respiratory virus infections are given in Table 1. 

**Table 1:** Seroprevalence of respiratory viral infections in great apes.

Virus	gorillas	orangutans	chimpanzees
	n = 77	n = 179	n = 305
RSV	79.3 ^#^ (61)	72.1 (129)	96.4 (294)
hMPV	46.8 (36)	10.1 (18)	42.6 (130)
inf A H3N2	3.9 (3)	5.6 (10)	11.2 (34)
inf A H1N1	3.9 (3)	19.0 (34)	71.5 (218)
infl B	58.4 (45)	75.4 (135)	26.2 (80)

^#^ Seroprevalence in percentages. Absolute numbers are given between brackets.

RSV was the most commonly found infection in the three ape species, with high frequencies of 72.1%, 79.3%, and 96.4% in orangutans, gorillas and chimpanzees, respectively. Other relatively common infections found in the apes were influenza B virus and human metapneumovirus infections. Orangutans presented the highest seroprevalence rate to influenza B virus (75.4%), while 58.4% of the gorilla sera contained antibodies against influenza B. In contrast, only 26.2% of the chimpanzee colony animals had antibodies to influenza B. A different infection pattern was seen for hMPV. Metapneumovirus infections were common in gorillas (46.8%) and the chimpanzee colony (42.6%), but the number of hMPV-seropositive orangutans was low (18 of 179 animals; 10.1%). Of the respiratory virus infections investigated in this study, antibodies to influenza A virus H3N2 were much less frequently detected in the various ape species. The highest seroprevalence (11.2%) was detected in sera from the chimpanzee colony, while very low numbers of seropositive sera were found in the gorillas (3.9%) and orangutans (5.6%). Equally low was the percentage of gorilla sera that reacted to the pandemic influenza H1N1 antigens, while 19% of the orangutan sera contained antibodies to H1N1. The latter figures differed considerably with those found in chimpanzee sera: more than 70% of the chimpanzee sera that were tested reacted positive to the H1N1 antigens. 

### 2.3. Respiratory Virus Infections in a Closed-Colony of Chimpanzees

The abovementioned figures for chimpanzees are based on the cumulative data from sera sampled during health checks spaced several years apart (1986, 1992, 1998, and 2000). Analysis of the data obtained from these four time points reveals remarkable changes in infection rates for the respiratory viruses (Table 2). Seroprevalence rates for RSV were high and relatively stable throughout the sampling period (84.6%, 100%, 98.1%, and 79.7%), but the rates for hMPV and the three influenza viruses declined between 1986 and 2000. Between 1986 and 1992, the number of seropositive animals to hMPV and influenza B virus started to decline. The percentage of animals with antibodies to hMPV decreased from 67.3% in 1986 to 42.5% in 1992, and further decreased to 21.2% and 21.7% in the subsequent sampling years. Influenza B virus infections decreased from 51% to 15%, and this more or less stabilized on this level in the following years (7.7% and 15.9%).

The percentage of chimpanzees with antibodies to influenza A virus H3N2 or H1N1 started to decrease between 1992 and 1998. For influenza A virus H3N2 percentages decreased from 17.5% in 1992, to 1.9% and 4.9% in 1998 and 2000, respectively. For the H1N1 virus, percentages decreased from 90% in 1992 to around 50% in the years 1998 and 2000. From 87 H1N1-seropositive animals, sera could be analyzed from multiple time-points. From 55 chimpanzees the median fluorescence intensity (MFI) signal decreased in time, including 23 animals that became negative for H1N1 antibodies at later time-points. Only two animals seroconverted between 1986 and 2000. RSV longitudinal analysis of sera revealed that between 1986 and 1992 the MFI increased in 89.3% of seropositive animals (25 out of 28 animals), between 1992 and 1998 in 46.7% (14/30), and in the period between 1998 and 2000 this figure was 36.1% (13/36).

**Table 2:** Longitudinal analysis of respiratory virus infections in a chimpanzee colony.

Virus	1986	1992	1998	2000
	n = 104	n = 80	n = 52	n = 69
RSV	84.6 (88)	100 (80)	98.1 (51)	79.7 (55)
hMPV	67.3 (70)	42.5 (34)	21.2 (11)	21.7 (15)
inf A H3N2	15.4 (16)	17.5 (14)	1.9 (1)	4.3 (3)
inf A H1N1	81.7 (85)	90.0 (72)	46.2 (24)	53.6 (37)
inf B	51.0 (53)	15.0 (12)	7.7 (4)	15.9 (11)

### 2.4. Introduction of Respiratory Viruses in a Group of Juvenile Chimpanzees

**Figure 1:**
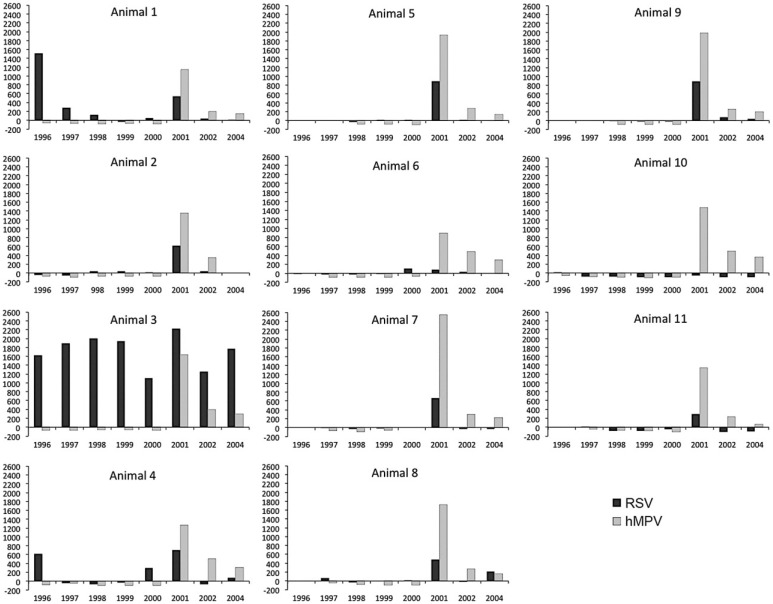
Serological screening of longitudinal serum samples from 11 juvenile chimpanzees using antigen-coated beads. On the horizontal axis the sampling date (in years) is indicated. On the vertical axis the median fluorescence intensity (MFI) is given for each serum sample assayed against respiratory syncytial virus (RSV) or human metapneumovirus (hMPV) antigens.

We furthermore investigated longitudinal sera of 11 young chimpanzees. According to the at that time current husbandry guidelines, babies were left with their mothers in a family group until they were at least 2 years old. Then, they were moved to a peer group of animals of the same age category, which was housed in a separate building. The eleven chimpanzees were followed for a period spanning 8 years. Antibodies to influenza A H3N2 virus were absent in the longitudinal sera, and low sero−reactivity was detected at irregular time-points in the sera of the young animals against influenza B virus and influenza A H1N1 virus antigens (data not shown). The antibody responses against RSV and hMPV are depicted in Figure 1. In the RSV assay, antibody titers to viral antigens were found in six animals early in infection, with signals varying to very low (#10 and #11), low (#8), to strong (#1, #3 and #4). The two animals with a clear antibody peak at the first sampling time−point, #1 and #4, were then 6 months and 1 month old, respectively. This makes it plausible that this early antibody peak was due to remaining maternal antibodies in the blood of these babies. Sera from animal #3 (15 months old at start of the observation period) contained significant levels of RSV antibodies during the whole observation period from 1996 to 2004, suggesting one or more newly acquired RSV infections in this animal. In nine animals, including chimpanzee #3, an antibody peak was seen in the sera collected in 2001, which points towards an introduction and spread of a RSV infection in the group of youngsters. The most clear-cut signals were obtained against hMPV. Antibody responses to this virus were completely absent in the animals until 2001. In that year responses suddenly peaked in all chimpanzees and were followed by a gradual decline in the subsequent years, indicating that hMPV was also introduced in the group in the one-year period between the 2000 and 2001 health checks. 

Interestingly, in the period a few months prior to the 2001 health check, the records kept by the animal caretakers twice mentioned an episode in which all animals showed symptoms of severe cold−like disease, including loss of appetite. Records also indicate that the animals were given cough medicine in their drinks.

## 3. Discussion

In free-ranging gorillas, chimpanzees, and bonobos, human metapneumovirus and flu-like viruses have been associated with morbidity and mortality in populations of these highly endangered ape species [19,20,21,22,23,24,25]. Inevitably, apes that are kept in (semi)captivity run an even greater risk of anthroponotic infections with pathogens from personnel working in primate-keeping institutes and, in case of zoos, from visitors. Viruses that can be transmitted via aerosolized droplets particularly pose a risk, as they do not rely on direct contact between individuals, and can travel considerable distances by air. 

We found serological evidence for infections with the human respiratory viruses in all three investigated ape species. In spite of the fact that we used a quite rigorous cut-off, we discovered high seroprevalence rates of selected human respiratory viruses in groups of (semi-) captive great apes, implying that the actual infection rates may be even higher in these animals. We also detected significant variations in seroprevalence rates between the different groups. This could point species−specific variations in susceptibility, but this may also be a consequence of differences in husbandry, housing conditions, or even the geographical location of the primate-keeping facility. 

Publications concerning anthroponotic infections of captive apes with respiratory viruses are scarce. The small group of 14 chimpanzees investigated by Kooriyama *et al.* [17] is comparable to the chimpanzees we investigated as they both have a laboratory background. The most striking discrepancy between groups is the high seropositivity to influenza A virus H1N1 found in the BPRC colony animals, while this infection is completely absent in the Kooriyama group. Beginning in 1990, BPRC implemented stricter guidelines to avoid anthroponotic and zoonotic infections, such as wearing facemasks, protective clothing, *etc.* This eventually led to a strong decrease in infection rates for most respiratory viruses (Table 2). Influenza A H1N1 seropositivity in 2000 was still significant (53.6%), but the analysis of longitudinal sera did reveal decreasing MFI signals in the majority of seropositive animals, suggesting a slow decrease in antibody titers. Possibly, the chimpanzees from the Japanese primate institute were kept under relatively more isolated conditions than the BPRC animals that were housed in outdoor enclosures. 

Chimpanzees are highly susceptible to infection with RSV. In fact, RSV was first isolated from a chimpanzee with coryza before this virus was recognized as a human pathogen [26]. Longitudinal analysis of sera from 36 animals showed that the number of new RSV infections was decreasing, but that nonetheless in the sera of 13 chimpanzees an increasing MFI signal was measured between 1998 and 2000. Additionally, new RSV infections were detected in several young chimpanzees (Figure 1). These findings suggest that RSV continued to circulate in the colony despite the stricter handling procedures. Novel anthroponotic RSV introductions cannot be fully excluded, but RSV circulating within a group of captive chimpanzees has been reported, and is thus another possible source of new infections [18].

Our analysis of longitudinal sera from young chimpanzees revealed that the different respiratory viruses do not display similar infection patterns. Over the 8-years observation period, influenza A and B viruses caused no, or sporadic infections in this group, and these scarce infections did not lead to a further circulation of the infection in the group. RSV and hMPV, on the other hand, both caused infections that rapidly spread in the whole group (hMPV), or to a majority of the animals (RSV). Why RSV and hMPV are more easily transmissible among chimpanzees than the influenza viruses remains obscure. Influenza A and B viruses use the same α2-6-linked sialic acid for binding to the cell surface, while the ubiquitous nucleolin molecule has been proposed as receptor for RSV [27,28]. The functional receptor for hMPV has not been conclusively characterized yet, but αvβ1 integrin and heparan sulfate have been suggested as candidates [29,30]. Receptor usage may explain the differences between influenza viruses and RSV/hMPV infection in chimpanzees, but does not clarify the high H1N1 seropositivity and the much lower rates for H3N2 and influenza B viruses in this great ape. In the latter case, amino acid sequence variations in the receptor-binding domain (RBD) of haemagglutinin may have influenced viral entry and the consequent infection. This domain, stretching from residue 63 to 286 [31] varies significantly. The RBD amino acid identities of the strains used in our antibody assay varied from 37% between H1 and H3, to 17% between the haemaglutinins of the influenza B strain and both influenza A strains.

Different infection patterns were also evident between the three ape species that cannot simply be explained by varying husbandry practices. Clearly, as the majority of orangutans have been confiscated after being illegally kept as pets, one can envision that they have been in close contact with humans and their pathogens. Yet, the seroprevalence for influenza A viruses and hMPV is relatively low, in contrast to the influenza B virus and RSV prevalences. During the annual influenza season influenza A and influenza B virus strains circulate alongside in the human population. Indeed, between 1992 and 1997 this was the situation in Europe and Asia: influenza A H3N2 and influenza B strains were the most prevalent in Europe and Asia, except for the 1995–1996 influenza season when influenza A H1N1 was dominant in Europe, and influenza A H3N2 was the most common influenza found in Asia (Weekly Epidemiological Records, World Health Organization). Consequently, the high number of influenza B virus infections in orangutans cannot be simply linked to prevailing influenza B viruses, as H3N2 strains were equally prevalent. Similarly, in the gorillas, that all originate from European zoos, influenza B virus was the second most common infection, after RSV, while influenza A virus reactivity was very low (3.9% for both H1N1 and H3N2). 

The above reasoning for respiratory viral infections in chimpanzees also applies for variations that were detected between species, but is not sufficient to explain why certain viruses predominate in one species. One can hypothesize that the different ape species are genetically predisposed to be more susceptible to specific viruses. Differences in expression levels of receptor molecules in airway epithelial cells in humans and great apes have been associated with the lesser sensitivity of chimpanzees to experimental infection with influenza A virus [32]. Moreover, several host cell factors can restrict virus replication after its binding to the cell receptor. Retrovirus replication can be restricted by APOBEC and TRIM5α host-cell proteins, and interferon responses can inhibit influenza A virus replication, and are often strain-specific [33]. 

Clearly, many questions regarding the inter- and intraspecies variations in infection patterns of respiratory viruses in great apes remain that warrant further research.

## 4. Experimental Section

### 4.1. Animals and Sera

Chimpanzee sera were obtained from the former colony of Western common chimpanzees (*Pan troglodytes verus*) that was housed until 2003 at the BPRC in Rijswijk, the Netherlands. Chimpanzees were housed in family groups in separate cages. Physical contact between animals from different cages was not possible, but because of the close proximity of the cages airborne transmission of viruses, or transmission via excreta or urine was conceivable. Sera were collected during the yearly routine health check of the colony animals. No animal was purposely bled for this study. All sera from Western lowland gorillas (*Gorilla gorilla gorilla*) had been sent to the BPRC for serology testing, and were used in this study. The sera from Bornean orangutans (*Pongo pygmaeus*) were collected for health purposes of routine health checks at Wanariset Orang-utan Rehabilitation Centre in East Kalimantan, Indonesia.

### 4.2. Multiplex Serology Testing

Immunoglobulin G class antibodies (IgG) targeted to human metapneumovirus (hMPV), respiratory syncytial virus (RSV), influenza A H3N2 virus (infA H3N2), pandemic influenza A H1N1 virus (infA H1N1), and influenza B virus (infB) were detected using an in-house developed magnetic bead-based multiplex assay. 

Virus preparations were obtained from different suppliers. An inactivated clarified cell lysate from hMPV was obtained from AbD Serotec (Düsseldorf, Germany). Inactivated influenza B virus (strain Hong Kong 5/72), influenza A H1N1 (strain California/7/2009), and influenza A H3N2 (strain Texas 1/77) were purchased from MyBioSource, Inc. (Emelca Bioscience, Breda, The Netherlands). RSV antigen (strain A2) was obtained from Advanced Biotechnologies Inc. (Tebu-bio BV, Heerhugowaard, The Netherlands).

Viral antigens were coupled to Bio-Plex Pro™ Magnetic COOH beads using the Bio-Plex Amine Coupling Kit (Bio-Rad Laboratories BV, Veenendaal, the Netherlands) according to the manufacturer’s instructions, but using 60 μg of protein per 1.25 × 10^6^ beads. A multiplex assay was set up and performed essentially as described by Kuller *et al.* [34], but optimized for use of magnetic beads. 

For a test, 2 × 10^3^ beads of each batch of coupled beads were mixed, and brought to a volume of 90 μL using StabilGuard^®^ BSA-free Immunoassay Stabilizer (Surmodics, Corporation, Eden Prairie, MN, USA). Then, 10 μL of serum was added (diluted 10 times in StabilGuard^®^), and incubated for 2 h at room temperature (RT) in the dark. Next, the beads were washed with PBS, mixed with 100 μL of biotinylated protein G (1:750 diluted in PBS) (Pierce Biotechnology, Rockford, IL, USA), and incubated 30 min in the dark at RT. After washing with PBS, 100 μL of 1:1000 diluted (in PBS) streptavidin-PE (Life Technologies Europe BV, Bleiswijk, The Netherlands) was added to the beads, followed by 10 min incubation at RT in the dark. Finally, the beads were washed 4 times with PBS, re-suspended in 125 μL PBS, and fluorescence was measured using a Bio-Plex^®^ 200 system (Bio-Rad Laboratories BV, Veenendaal, The Netherlands). The assay was established using human sera that were mono-specific to the respiratory viruses investigated in this study. Aliquots of each batch of coupled beads were mixed and incubated with each serum, and with a pool of the sera. Clear−cut signals were obtained when testing the right antigen-serum combination, while the background signal with other antigens remained below the signal obtained with a negative control serum. Performance of the bead-based assay was evaluated using Western blot as the gold standard. Blots were made with the virus preparations used for coupling to the magnetic beads. As set of twenty chimpanzee sera was used to validate the assay. The overall sensitivity of the assay was 94% (37/43), and the specificity was 86% (63/67). For the individual viruses, sensitivity and specificity for RSV, infA H3N2 and hMPV was very high. For RSV sensitivity and specificity were 94% (16/7) and 100% (4/4), for infA H3N2 100% (11/11) and 100% (9/9), and for hMPV 94% (15/16) and 100% (5/5). For the infA H1N1 and infB viruses the sensitivities and specificities were somewhat less; both assays showed three false-positive outcomes in the bead-based assay, and one false-negative result. The percentages sensitivity and specificity were 91% (10/11) and 77% (10/13) for infA H1N1, and for inf B 92% (11/12) and 75% (9/12).

### 4.3. Data Analysis

The sera were assayed in a 96-well format that included 88 test sera, two positive human control sera, two PBS controls, and four negative control sera obtained from chimpanzees. The positive control sera and the negative sera had been screened in a hospital diagnostic laboratory for presence or absence of antibodies to the viruses used in this study. All sera were tested in duplicate. The median fluorescence intensity (MFI) was determined from a minimum of 50 counted events per batch of antigen-coupled beads. Sera were considered positive when the MFI signal was higher than four times the average background signal. The background signal was the average MFI of duplicate analysis of the four negative sera.

## 5. Conclusions

In this study we have confirmed the sensitivity of great ape species to human respiratory viruses. Our findings thus support the measures taken to reduce the threat of disease transmission from tourists and researchers to the highly endangered Virunga mountain gorillas. The prohibition for tourists and researchers to stay at least seven meters away from the gorillas and to wear surgical masks has led to a decrease in the incidence of respiratory viral diseases [35]. 

Respiratory viruses can cause clinical symptoms in captive chimpanzees, like coughing, sneezing and loss of appetite, and infections can spread rapidly in colonies of captive animals. In contrast to the reported mortalities amongst wild apes, respiratory virus infections in captive apes generally seem to have a relatively mild disease course. This was seen in the group of juvenile chimpanzees described in this study, but also reported by Kooriyama *et al.* [17]. For captive apes, the implementation of strict guidelines for handling and housing of nonhuman primates has shown to be efficient in the reduction of novel infections on colonies of captive animals. 
